# Interventions to Improve Patient Care on Surgical Ward Rounds: A Systematic Review

**DOI:** 10.1007/s00268-023-07221-z

**Published:** 2023-10-19

**Authors:** Reuben He, Sameer Bhat, Chris Varghese, Jeremy Rossaak, Celia Keane, Wal Baraza, Cameron I. Wells

**Affiliations:** 1 Department of Surgery, Faculty of Medical and Health Sciences, Surgical and Translational Research Centre, The University of Auckland, Private Bag 92019, Auckland, New Zealand; 2 Department of Surgery, Te Whatu Ora MidCentral, Palmerston North, New Zealand; 3 Department of General Surgery, Tauranga Hospital, Te Whatu Ora Bay of Plenty, Tauranga, New Zealand; 4 Department of General Surgery, Te Whatu Ora Te Toka Tumai Auckland, Auckland, New Zealand

## Abstract

**Background:**

Ward rounds are an essential component of surgical and perioperative care. However, the relative effectiveness of different interventions to improve the quality of surgical ward rounds remains uncertain. The aim of this systematic review was to evaluate the efficacy of various ward round interventions among surgical patients.

**Methods:**

A systematic literature search of the MEDLINE (OVID), EMBASE (OVID), Scopus, Cumulative Index of Nursing and Allied Health (CINAHL), and PsycInfo databases was performed on 7 October 2022 in accordance with PRISMA guidelines. All studies investigating surgical ward round quality improvement strategies with measurable outcomes were included. Data were analysed via narrative synthesis based on commonly reported themes.

**Results:**

A total of 28 studies were included. Most were cohort studies (*n* = 25), followed by randomised controlled trials (*n* = 3). Checklists/proformas were utilised most commonly (*n* = 22), followed by technological (*n* = 3), personnel (*n* = 2), and well-being (*n* = 1) quality improvement strategies. The majority of checklist interventions (*n* = 21, 95%) showed significant improvements in documentation compliance, staff understanding, or patient satisfaction. Other less frequently reported ward round interventions demonstrated improvements in communication, patient safety, and reductions in patient stress levels.

**Conclusions:**

Use of checklists, technology, personnel, and well-being improvement strategies have been associated with improvements in ward round documentation, communication, as well as staff and patient satisfaction. Future studies should investigate the ease of implementation and long-term durability of these interventions, in addition to their impact on clinically relevant outcomes such as patient morbidity and mortality.

**Supplementary Information:**

The online version contains supplementary material available at 10.1007/s00268-023-07221-z.

## Introduction

Ward rounds are an essential component of surgical and perioperative care [1]. They allow doctors to communicate with patients, assess progress, and develop treatment plans [2]. The quality of ward rounds may directly impact on patient outcomes [3, 4], with documentation being a key method of communication between clinical teams [1, 5].

Regulatory bodies have provided expected standards of communication and documentation in doctor-patient consultations [6]. Multiple studies have found that documentation during surgical ward rounds consistently fails to achieve these standards [3, 4].Shortfalls may lead to delays in diagnosis, precipitate preventable complications, medicolegal challenges, and ultimately result in worse outcomes for patients [1, 7, 8].

Ward round checklists and proformas have been developed in an attempt to improve patient care through better documentation of patient progress and management plans [9–12]. Studies have demonstrated improvements in perioperative care through reductions in rates of error and failure to rescue (i.e. death after the development of a postoperative complication), when ward round checklists were utilised [11, 12]. Telerounding and the use of bedside nursing summaries have also been suggested as adjuncts to the standard ward round for surgical patients [13, 14].

Current literature demonstrates a wide variety of different interventions to improve the quality of surgical ward rounds [9, 13–15]. However, there is uncertainty surrounding their relative effectiveness, ease of implementation, and impact on patient satisfaction. The objectives of this study were to systematically review and assess the efficacy of previously documented interventions. This may aid in the design and implementation of perioperative quality improvement strategies.

## Methods

The protocol for this review was prospectively registered on PROSPERO (ID: CRD42022359414) [16]. The review complied with the Preferred Reporting Items for Systematic Reviews and Meta-Analyses (PRISMA) 2020 guidelines (refer Supplementary Appendix S1 for the PRISMA checklist) [17].

## Data sources and search strategy

A systematic literature search of the MEDLINE (OVID), EMBASE (OVID), Scopus, Cumulative Index of Nursing and Allied Health (CINAHL), and PsycInfo databases was performed 7 October 2022. The search string consisted of key words and Medical Subject Headings (MeSH) terms for various surgical specialties (e.g. ‘cardiothoracic’, ‘otorhinolaryngology’, ‘vascular’), medical staff members (e.g. ‘attending’, ‘consultant’, ‘registrar’), and ward rounds (e.g. ‘ward round’, ‘bedside round’, ‘morning round’), among others. These terms were combined using the ‘AND’/‘OR’ Boolean operators (refer to Supplementary Appendix S2 for an exemplar search string using the MEDLINE database).

Databases were searched from their date of inception. The results were restricted to studies published in English. There were no limitations on patient age, geographic location, or study design. Reference lists of included studies and relevant systematic reviews were also hand-searched to identify additional studies for inclusion.

## Study selection criteria

All original studies investigating quality improvement strategies implemented during an inpatient surgical ward round were eligible for inclusion. Surgical ward round was defined as any setting or situation in which member(s) of a surgical team assessed patients as part of perioperative care, regardless of surgical specialty. Only studies reporting quality improvement interventions with a measurable outcome on an individual patient (e.g. patient satisfaction, understanding, and/or interpretation of quality of care) or hospital/department (e.g. duration of ward round, time spent per patient, documentation completion rate, and/or percentage of clinical information considered), and those where the majority (> 50%) of included patients were under surgical care, were included.

We excluded case reports (with< 5 patients), articles without an accessible full-text and/or conference abstracts without a full-text publication. Reviews and studies published in languages other than English were also excluded.

## Screening process

Article records from the database searches were exported into EndNote X9 (Clarivate, Philadelphia, PA, USA) and de-duplicated using validated methods [18]. Two investigators (RH, SB) used the Rayyan web application to independently screen titles and abstracts, with relevant full texts then considered for final inclusion [18, 19]. Any discrepancies were addressed through discussion with input from a senior author (CW), until consensus was reached.

## Data extraction

Relevant data from included studies were extracted into a proforma Google spreadsheet by a single investigator (RH). These data were independently validated by a second investigator (SB), with any disagreements resolved via mediation with a senior investigator (CW) until consensus was reached. Extracted data comprised study characteristics, conflicts of interest, study funding, surgical specialty, number and designation of medical staff involved, sample size (pre and post-intervention), description of intervention and method of implementation, as well as the comparator intervention. Individual patient and/or hospital/departmental level outcomes were also extracted. Data that were reported in the form of graphs and/or figures were extracted using WebPlotDigitizer (version 4.5; Pacifica, California, USA) [20]. Attempts were made to contact corresponding authors in cases of ambiguous or missing data [21].

## Quality assessment

Two authors (RH and SB) independently performed methodological quality assessment of included studies, with disputes resolved through discussion. The Risk of Bias in Non-Randomized Studies of Interventions (ROBINS-I) tool [22] and Joanna Briggs Institute (JBI) Critical Appraisal Checklist [23] were used to appraise prospective and retrospective cohort studies, respectively, while the Cochrane Collaboration’s Risk of Bias 2.0 (ROB2) tool was used to assess risk of bias within randomised controlled trials (RCTs) [24]. ROBINS-I results were depicted pictorially using the Risk-of-Bias Visualization (robvis) package in RStudio (R Studio, Boston, MA) [25].

## Analysis

Data were analysed via narrative synthesis according to major reported themes among the included studies. Simple descriptive statistics were used to quantitatively report data where possible.

## Results

### Search results

**Fig. 1:**
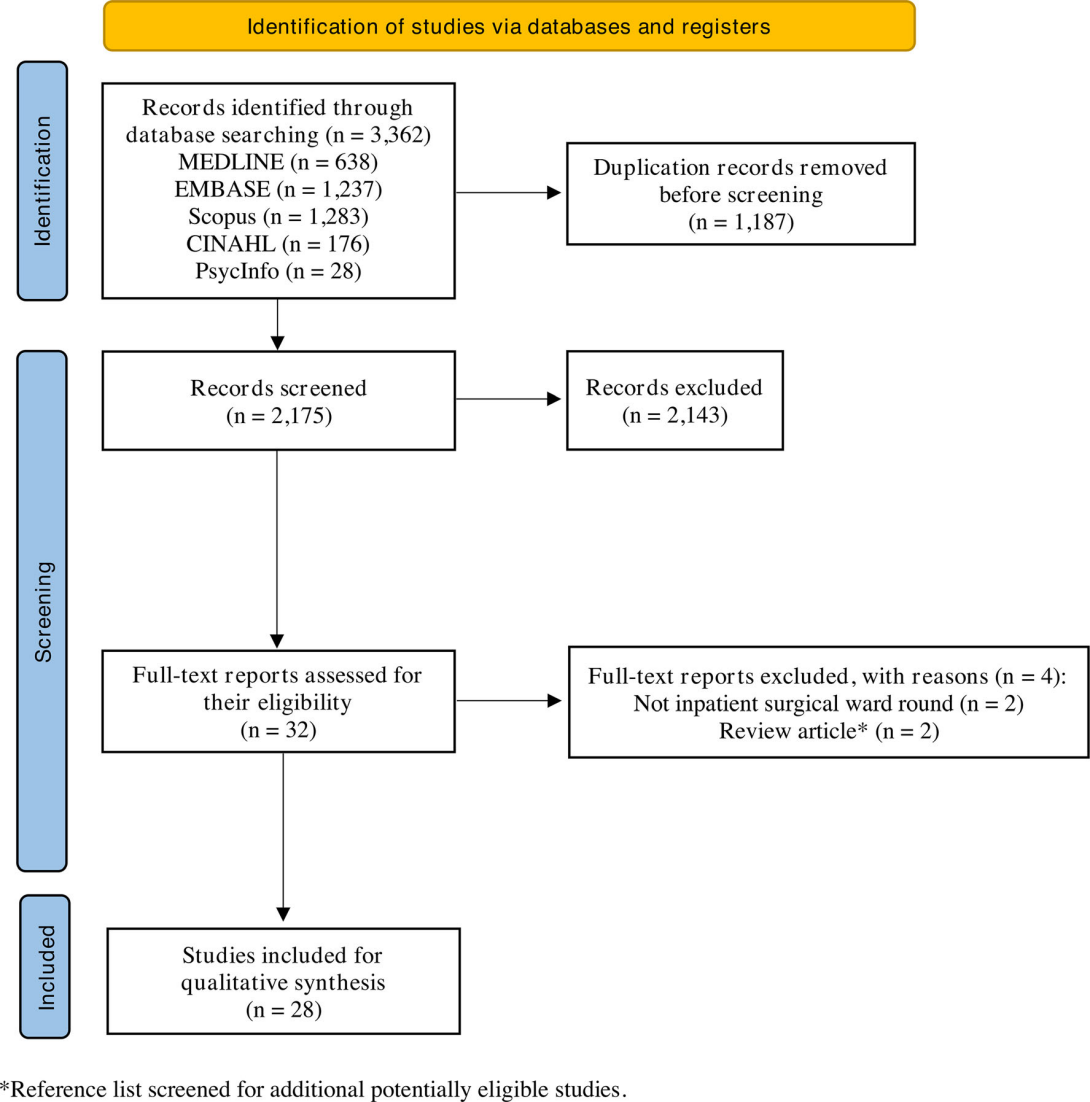
Preferred reporting items for systematic reviews and meta-analyses (PRISMA) flow diagram showing the selection process for studies included in the systematic review

The database search yielded a total of 3362 results, from which a total of 28 studies were included in the qualitative synthesis (Fig. 1) [3, 5, 9, 12–14, 21, 26–46].

## Study characteristics

Characteristics of the 28 included studies are provided in Table 1. Other than three studies, the remainder were published within the last decade [21, 31, 35]. Most were cohort studies (*n* = 25, 89.3%), followed by RCTs (*n* = 3, 10.7%). Most studies were conducted in the UK (*n* = 11, 39.3%), followed by the USA (*n* = 6, 21.4%), and Aotearoa New Zealand (*n* = 4, 14.3%). All were single-centre studies, including patients from a range of surgical specialties; general (*n* = 8, 28.6%) and orthopaedic surgery (*n* = 6, 21.4%), surgical intensive care unit (including general surgical, trauma, and burns patients; *n* = 3, 10.7%), trauma surgery (*n* = 3, 10.7%), and urology (*n* = 3, 10.7%) were the specialties assessed most frequently.

**Table 1:** Characteristics of studies included in the review

First author (year)	Intervention	Study design (R/P)	Country	Study period	Surgical specialty	No. and designation of medical staff	Sample size (pre-, post-intervention)	Conflicts of interest
Abbas (2016)	‘Surgeon of the week’ rounding system	Cohort study (R)	USA	July–December 2012 (pre-intervention); July–December 2013 (post-intervention)	Paediatric Surgery	Paediatric surgical consultants (n = 15)	2356, 2837	None
Al-Mahrouqi (2013)	Post-acute ward round proforma/checklist	Cohort study (R)	NZ	May 2012 (pre-intervention); November 2012 (post-intervention)	General Surgery	General surgical consultants (n = 5 or 6), with a varied number of registrars and junior house surgeons	108, 103	None
Alamri (2016)	Ward round checklist/proforma	Cohort study (R)	NZ	July 2015	General Surgery	General surgical consultants (unspecified number) and other junior staff (registrars and house surgeons; unspecified number)	N/A, 103	NS
Alazzawi (2016)	Ward round checklist/proforma	Cohort study (R)	UK	January–June 2015	Trauma and Orthopaedics	Trauma and orthopaedic registrars (n = 2)	20, 20	NS
Armas (2021)	Active/scheduled breaks during ward rounds	Cohort study (P)	USA	October–December 2019	Surgical ICU^*^	Consultant (n = 1), fellow (n = 1), residents (n = 2), interns (n = 1 to 2), nurse (n = 1), physician assistant (n = 1), medical students (n = 1 to 4)	N/A, 30	None
Aydogdu (2019)	Additional telerounding on patients following surgery	RCT (P)	Turkey	Not stated	Urology	Urology consultant (n = 1)	40, 40	NS
Baker (1986)	Presence of a radiologist during ward rounds	Cohort study (R)	USA	March 1983–June 1984	General Surgery	Consultant radiologist (n = 1), supervising general surgical consultant (n = 1), surgical registrars, house officers and medical students (unspecified numbers)	721, 765	NS
Banfield (2018)	Post-acute ward round proforma/checklist	Cohort study (R)	UK	April 2014 (pre-intervention); June 2014, April 2015, and February 2017 (post-intervention)	General Surgery	General surgical consultant (n = 2), house surgeons (unspecified number), and senior SAU nurse (n = 1)	31, 97	Senior author (SKR) also co-authored a study which included the Royal United Hospital Foundation NHS (study centre) as one of the participating centres in the Emergency Laparotomy Pathway Quality Improvement Care Study
Blucher (2014)	Ward safety proforma/checklist	Cohort study (R)	Australia	NS	General Surgery	Junior surgical staff (number and designation not specified)	49, 51	NS
Brown (2019)	Surgical communication check sheet/proforma	Cohort study (P)	UK	October 2016–April 2017	Trauma and Orthopaedics	Consultant surgeon (n = 1), orthopaedic research fellow (n = 1), FY-2 junior doctor (n = 1), medical students (n = 2)	170, 111	Senior author is a paid consultant for Stryker (R + D and Education), as well as an educational consultant for Smith and Nephew (London, UK) and Orthofox (Texas, USA)
Byrnes (2009)	Ward round checklist/proforma	Cohort study (R)	USA	June 2006–May 2007	Surgical ICU ^?^	SICU consultant, fellow, house surgeons, nurses, pharmacist (n = 1), and dietitian (n = 1)	583, 671	None
Chaudary (2022)	Digital/electronic patient records	Cohort study (R)	UK	February–August 2021	Trauma and Orthopaedics	Consultants (n = 5), registrars (n = 4), senior house surgeons (n = 13), nurses (n = 14), and clinical support workers (n = 4)	44, 44	None
Crowson (2016)	Mobile tablet use during ward rounds	Cohort study (P)	USA	NS	Otorhinolaryngology	Registrars or house surgeons (PGY1 to 5; n = 13)	13, 13	None
Dhillon (2011)	Ward round checklist	Cohort study (P)	Ireland	NS	General Surgery, Vascular Surgery, Plastic Surgery, Neurosurgery	Consultants (n = 5)	53, 34	NS
Dolan (2016)	Post-take ward round checklist/proforma	Cohort study (P)	UK	NS	NS	Consultants (number not specified)	50, 47	None
Duxbury (2013)	Post-take ward round checklist/proforma	Cohort study (P)	Unclear	NS	Trauma and Orthopaedics	Consultants, registrars, and junior house surgeons (unspecified number)	50, 50	None
Gilliland (2018)	Ward round template/checklist	Cohort study (R)	UK	NS	Urology	Rounding team (number and designation not specified)	Unspecified, 45	None
Koumoullis (2020)	Surgical Tool for the Assessment of Rounds (STAR) checklist/proforma	Cohort study (R)	UK	September–December 2017	Plastic Surgery	Rounding team including junior house surgeons (number and designation not specified)	42, 103	None
Krishnamohan (2019)	Ward round checklist	Cohort study (P)	UK	April 2015–August 2016	Urology and Vascular Surgery	Rounding team (number and designation not specified)	72, 61	None
Ng (2018)	Ward round sticker/checklist	Cohort study (R)	UK	December 2016–March 2017	General Surgery	Senior general surgical registrar (*n* = 1), senior house surgeons (*n* = 2), FY-1 junior doctor (n = 2)	109, 147	None
Pitcher (2016)	Ward round checklist	Cohort study (R)	Australia	NS	General Surgery	Consultant (n = 1), registrars (n = 6), house surgeons (*n* = 3)	132, 182	NS
Pucher (2014)	Ward round checklist	RCT (P)	UK	NS	General Surgery	General surgical registrars (*n* = 20), junior house surgeon (*n* = 1), nurse (*n* = 1), medical actors [as patients] (*n* = 3)	10, 10	None
Read (2021)	Ward round checklist	RCT (P)	NZ	NS	NS	Consultants (unspecified number)	68, 56	None
Shaughnessy (2015)	Ward round checklist	Cohort study (P)	UK	NS	Cardiothoracic Surgery	Anaesthetists (*n* = 9), rounding team (designation and number not specified), bedside nurses (number not specified)	162, 83	NS
Talia (2017)	Ward round checklist	Cohort study (P)	Australia	NS	Orthopaedics	Junior house surgeons (*n* = 4)	132, 68	None
Tranter-Entwistle (2020)	Ward round checklist	Cohort study (P)	NZ	NS	Vascular Surgery	Rounding team consisting of: house officer, clinical nurse specialist, registrars, SMO, physiotherapist, dietitian, social worker, occupational therapist, and attending nurse (numbers not specified), as well as a final year medical student (*n* = 1)	60, 173	None
Yorkgitis (2018)	Laboratory tests and chest X-ray imaging section on daily ICU checklist	Cohort study (R)	USA	July–October 2015 (pre-intervention); October 2015–January 2016 (post-intervention)	Surgical ICU ^??^	Surgical ICU staff, including: anaesthetists, ED clinicians, surgical residents, and surgical critical care fellows (number not specified)	155, 152	None
Vukanic (2021)	Ward round proforma	Cohort study (R)	Ireland	November 2017 −March 2018	Orthopaedics	Rounding team consisting of an SMO (*n* = 1) and junior staff (not further specified)	30, 30	None

ICU, intensive care unit; NHS, National Health Service; NS, not stated; NZ, New Zealand; P, prospective; R, retrospective; RCT, randomised controlled trial; UK, United Kingdom; USA, United States of America; SMO, senior medical officer; ED, emergency department

* Inclusive of general surgical and patients from other surgical subspecialties (not further specified) who were managed in the surgical ICU

^?^ Inclusive of trauma and general surgical patients who were managed in the surgical ICU

^**??**^ Includes general surgical, trauma, and burns patients managed in a surgical ICU

## Quality assessment

Quality assessment results using the ROBINS-I tool are depicted in Supplementary Figure S1**.** Four prospective cohort studies were judged to be at critical risk of bias, principally due to the impact of unmeasured confounding variables [13, 30, 34, 38] Outcomes were measured through valid and reliable means, with sufficiently long follow-up duration, in 12 of the 15 (80%) retrospective cohort studies (Supplementary Appendix S3). However, none of the authors identified or statistically adjusted for any confounding factors in their analyses. The RCTs were mostly at high risk of bias (*n* = 2 studies, 66.7%) [9, 14], resulting from outcome assessors who were unblinded to the ward round intervention of interest (Supplementary Figures S2–3).

## Ward round interventions

A range of quality improvement interventions were implemented during surgical ward rounds. In total, 22 studies used some form of a ward round checklist or proforma (refer to Supplementary Appendix S4 for an example of a ward round checklist). Other interventions included a ‘surgeon of the week’ rounding system (*n* = 1) [26], additional telerounding on postoperative patients (*n* = 1) [14], involvement of a specialist radiologist during the ward round (*n* = 1) [31], digital record keeping (*n* = 1) [36], mobile tablet use during inpatient services (*n* = 1) [37], and implementation of active breaks during the ward round (*n* = 1) [30].

Checklists and proformas served as a guidance for information that should be covered in a surgical ward round, or a template to ensure adequate documentation of essential ward round points. Of the 22 studies that employed a checklist or a proforma, most introduced physical stickers or forms which were placed in a patient’s medical record (82%, 18/22), whereas information printouts displayed throughout the ward were trialled in three studies (Table 2) [34, 44, 46].

**Table 2:** Summary of findings and limitations of included studies utilising a ward round checklist or proforma

First author (year)	Intervention	Method of implementation	Improved documentation/adherence	Patient satisfaction	Staff satisfaction	Limitations
Al-Mahrouqi (2013)	Post-acute ward round proforma/checklist	Standardised ward round proforma introduced as a sticker attached to a patient’s notes, and implemented for 6 months prior to post-intervention data collection	Improvement in documentation of time and date (37% vs 72%) and impression (40% vs 61%); improvement in documentation of dietary plan when proforma filled out (78/103 patients, 76%	N/A	No statistically significant impact on nurse certainty of dietary plan and number of times needed to contact surgical teams	Contamination from nurses discussing study; lack of complete documentation on post-acute consultant ward round; low maintenance of intervention (75% proforma usage 6 months post-intervention); poor survey response rate
Alamri (2016)	Ward round checklist/proforma	Checklist implemented during inpatient surgical ward rounds	Most fields in proforma documented to adequate level (> 80% documentation) 2 years post-intervention	N/A	N/A	Timing bias, ‘snapshot’ vs longitudinal study; lack of exploration of freehand notes to identify reasons for proforma documentation deficiency
Alazzawi (2016)	Ward round proforma/checklist	Two versions (1. tickbox; 2. white spaces) of ward round checklist utilised, with a training session provided before implementation of each version. Each version was trialled for a period of 7 days, with a minimum 2-week gap between the trial of versions 1 and 2	Significant increases in documentation of diagnosis and management, objective assessments (excluding observations noted), and logistics	N/A	10 members of staff all preferred proforma vs standard care due to ease of reading and clarity of information	Effect on clinical assessment and patient care not measured; unblinded study; large amount of undocumented clinical activity
Banfield (2018)	Post-acute ward round proforma/checklist	Ward round checklist consisting of 10 different points, to be used as a ‘time out’ after each patient with clarification of these points from the whole surgical team	Improvement in documentation of VTE assessment, fluids, observations and investigations post-intervention; improved weekend documentation in all categories except length of stay	N/A	junior team members found that checklist improved understanding of diagnosis, management plan, and ward round effectiveness	Small sample size; reduced checklist access for outlying patients
Blucher (2014)	Ward safety proforma/checklist	Junior surgical staff formally educated on ward safety checklist, with implementation for 1 week during surgical ward rounds	Overall significant improvement in introduction phase components of checklist (31% vs 52%); overall significant improvement in time-out phase components (37% vs 45%); overall significant improvement in actions phase components (48% vs 56%)	N/A	N/A	Small sample size; no standardisation of time-out phase components in checklist; effect on clinical assessment and patient care not measured
Brown (2019)	Surgical communication check sheet/proforma	Ward round checklist comprising of 13 questions, including a mixture of yes/no questions and 10-point Likert scale questions (very poor —> excellent), which were employed during the trauma ward round	N/A	Reduction in percentage of patients with unanswered questions (21.8% vs 16.7%), reduction in number of patients unsure why a test was done (25.9% vs 12.7%), improvement in average understanding of management plan (64.7% to 83.3%)	N/A	Study unblinded; reduced sample size (survey compliance issues)
Byrnes (2009)	Ward round checklist/proforma	All SICU consultants and fellows were educated and encouraged to use the checklist during morning ward rounds	Verbal consideration of domains improved from 90.9% to 99.7% after intervention	N/A	N/A	Contamination bias in consideration phase (as checklist was optional for both groups); observer bias; no quantifiable data for some domains on checklist (e.g. tracheostomy protocol, need for central venous catheter, nutrition); questions about longitudinal checklist maintenance
Dhillon (2011)	Ward round checklist	Consultants were educated on the importance of ward round handovers and the use of the ward round checklist	Improvement in percentage adherence to the Good Surgical Practice Guidelines (55% vs 91%); significant improvement in documentation across all areas measured	N/A	N/A	Did not measure effect on morbidity and mortality; Hawthorne effect;
Dolan (2016)	Post-take ward round checklist/proforma	Information about ward round proforma disseminated via email; each admitted patient had a form placed in their admission documentation, and proforma was used for each post-take ward round	Improvement in documentation compliance across multiple categories	N/A	N/A	Small sample size; unblinded (Hawthorne effect)
Duxbury (2013)	Post-take ward round checklist/proforma	Proforma written on yellow paper which was placed in the patient’s notes	Improvements in documentation of multiple categories:	N/A	N/A	Small sample size; poor compliance to checklist during weekends, unblinded
Gilliland (2018)	Ward round template/checklist	Three Plan-Do-Study-Act (PDSA) cycles were performed to implement the new ward round template; changes were iteratively made to the ward round template based on results and further discussion after each cycle was implemented	Significant improvements in documentations of VTE risk assessment (14% to 92%) and antibiotic stewardship (0% to 100%), and use of the treatment escalation plan form (29% to 78%)	N/A	N/A	Small sample size; patient outcomes not measured, assumption of association between improved documentation and improved patient outcomes
Koumoullis (2020)	Surgical Tool for the Assessment of Rounds (STAR) checklist/proforma	STAR tool implemented during daily ward rounds	Checklist implementation improved STAR completion rate (47% to 70% to 88%);	N/A	Unsolicited enthusiastic staff comments about ward round improvement after STAR implementation	Hawthorne effect, weekend exclusion, seasonal patient variation
Krishnamohan (2019)	Ward round checklist	Checklist printed on yellow labels which were placed in patient clinical notes for documentation during the daily ward round	Overall documentation of six checklist parameters improved following implementation (26% to 79%); 3-month follow-up showed maintenance of 72% documentation compliance	N/A	N/A	Checklist reporting bias; quality of documentation not assessed; Hawthorne effect; relevance to patient outcomes not measured
Ng (2018)	Ward round sticker/checklist	Ward round stickers were placed in a patient’s notes, followed by review of sticker compliance	Significant improvement in checklist adherence across multiple tasks	N/A	N/A	Relevance to patient outcomes not measured; data for outlying patients not collected; Hawthorne effect
Pitcher (2016)	Ward round checklist	Ward round completed with a member of the team as a ‘prompter’ to encourage checklist criteria coverage	Significant improvement in the consideration of the majority of checklist criteria	N/A	N/A	Hawthorne effect (surgical team blind to nature of observations but were aware that observation was being conducted)
Pucher (2014)	Ward round checklist	Checklists implemented during daily wards, and adherence to critical care processes assessed in addition to technical and non-technical skills	Intervention group subjects using checklist had significantly fewer critical errors compared with controls (median(i.q.r.) 0(0–0) vs 60(40–73)%		Subjective ease of checklist use	Did not measure checklist use for medical staff outside of surgical trainees; single-centre study; did measure maintenance of checklist over time;
Read (2021)	Ward round checklist	Checklist implemented during the daily ward round	Overall percentage of checklist items endorsed increased significantly after intervention (64.8% to 70.0%)	N/A	N/A	Small sample size; patient could not compare standard vs checklist-implemented ward rounds as only subjected to one or the other; poor compliance with checklist completion from surgical teams; Hawthorne effect
Shaughnessy (2015)	Ward round checklist	Ward round checklist implemented during the daily ward round	87% of MDT respondents noticed improvement in bedside nurse attendance during ward round	N/A	97% of nurses agreed that verbal checklist summarising improved clarity and 90% felt it improved patient care	Patient understanding of ward round not measured; large variation in pre- vs post-checklist observation numbers—time limitation of post-audit; difficulty enforcing nurse checklist review compliance
Talia (2017)	Ward round checklist	Checklist implemented during the daily ward round	Significant improvement in documentation across multiple categories	N/A	N/A	Variation in pre- and post-checklist sample sizes; did not measure impact on patient outcomes
Tranter-Entwistle (2020)	Ward round checklist	Checklist implemented during the daily ward round	20/21 ward round quality indicators showed statistically significant improvement after checklist implementation	N/A	N/A	Lack of external checklist validation; single centre; single observer; no measure of impact on patient outcomes
Yorkgitis (2018)	Laboratory tests and chest X-ray imaging section on daily ICU checklist	Implementation of the checklist during the daily ICU ward round	No statistical reduction in laboratory tests or chest x-ray imaging ordered per day after checklist implementation	N/A	N/A	Checklist fatigue; checklist not reviewed daily;
Vukanic (2021)	Ward round proforma	Ward round proforma implemented during the daily ward round	After proforma introduction, average documentation criteria fulfilment percentage increased (0% to 86%); maintenance was 75% criteria fulfilment after 2 months	N/A	N/A	Small sample size; baseline data collected on single day

SMO, senior medical officer; FY, foundation year; SICU, surgical intensive care unit; ICU, intensive care unit; CT, computerised tomography; VTE, venous thromboembolism; DVT, deep vein thrombosis; PTWR, post-take ward round; MDT, multi-disciplinary team

## Outcomes

Main findings and limitations of included checklist/proforma studies are summarised in Table 2. Supplementary Appendix S5 provides a summary of findings and limitations of all included studies grouped by theme of intervention.

### Documentation criteria

Four studies implemented a ‘Plan, Do, Study, Act’ (PDSA) cycle design, whereby ward round interventions were iteratively reviewed and improved after each study [5, 13, 27, 32]. Proforma checklists were used in all of these, in addition to completion of a pre-intervention audit to evaluate baseline documentation compliance against agreed documentation criteria. All studies demonstrated significant improvements in most criteria, such as the documentation of date and time, clinician leading ward round, impression, management plan, and venous thromboembolism (VTE) assessment. Alamri and colleagues [28] reviewed compliance against a proforma sticker utilised in a previous study [27]

### Resources and personnel

Yorkgitis et al*.* [46] introduced a laboratory tests and chest X-ray imaging section on their daily intensive care unit (ICU) checklist. There was no significant difference in the mean number of chest x-rays and coagulation tests requested each day. There was also no significant change in the mean daily number of complete blood counts, chemistry panels, arterial blood gases, and red blood cell transfusions ordered.

Baker et al*.* [31] reported that presence of a consultant radiologist on the surgical ward round resulted in a significant reduction in the number of nuclear medicine scans, ultrasound scans, body computed tomography (CT) scans, barium enemas, and upper gastrointestinal (GI) series performed. The average hospital length of stay also decreased from 21.4 to 18.4 days. Interestingly, the number of abdominal plain films obtained increased when a consultant radiologist was present.

### Staff and patient satisfaction

Pre- and post-intervention surveys were completed by staff and patients to measure satisfaction levels. Generally, ward round quality improvement strategies were well received by staff and patients. Two studies found that checklists had utility as a tool for learning and guiding ward round documentation [3, 21]. Krishnamohan et al. [3] found ward round checklists to be a useful method for deconstructing power hierarchies and encouraging junior team members to ask questions regarding patient care.

Non-checklist interventions also elicited positive responses. Interventions such as the institution of active breaks during the surgical ward round [30], adjunctive telerounding [14], and use of electronic patient records [36] all demonstrated improved staff satisfaction compared to standard surgical ward rounds. In addition, Chaudary and colleagues [36] explored how electronic patient records created extra opportunities for junior staff to learn imaging interpretation techniques amidst the ward round. Abbas et al. [26] concluded that a ‘surgeon of the week’ rounding system was beneficial for both staff and patient satisfaction, and also patient safety and efficiency of the surgical ward round. Following implementation, there were a reduction in the total number of safety complaints, an increase in work relative value units/revenue, and an increase in both employee satisfaction and parental satisfaction in a paediatric surgical unit.

### Communication and documentation

Five studies investigated the impact of checklist interventions on communication between staff and patients. Alazzawi et al. [29] reported that all surveyed staff members (*n* = 10) preferred a proforma to standard ward rounds due to improved clarity of information. Banfield et al. [32] demonstrated improvements in communication and understanding of diagnosis and management plans among junior team members when a proforma was used during the post-acute surgical ward round. Brown et al. [34] observed improvements in patient understanding of their management plans when a surgical communication checksheet was used. Al-Mahrouqi et al*.* [27] demonstrated that although improvements in ward round documentation were seen with a post-acute ward round proforma, there was no statistically significant impact on nurse certainty of dietary plans, and the number of times surgical teams were contacted. Shaughnessy and colleagues highlighted that patient communication required further improvement, despite a verbal checklist demonstrating improved nursing clarity and reduced plan omissions being used [13]

### Surgical ward round efficiency

Significant reductions in overall ward round duration were observed through the use of mobile tablet technology [37] and a ward round checklist [21, 38]. Aydogdu et al*.* [14] found that adjunctive telerounding did not result in a statistically significant difference in mean ward round time which was consistent with two other studies that employed a ward round proforma [44, 45].

### Patient outcomes

Only two studies investigated the impact of ward round interventions on perioperative patient outcomes [1, 3, 14]. Krishnamohan et al. [3] identified that use of a ward round checklist reduced errors in medication prescriptions, antimicrobial administration, fluid balance monitoring, patient observation charts, and the number of venous thromboembolism (VTE) cases diagnosed. Pucher et al. [1] found that general surgery trainees who utilised a ward round checklist committed significantly fewer critical errors compared to standard surgical rounding, with critical errors defined as the ‘failure to adhere to critical processes in the management of postoperative complications’.

### Resilience

Few studies described the durability of surgical ward round quality improvement strategies [3, 27, 28]. Results were inconsistent in two studies; Al-Mahrouqi et al*.* found that compliance was low six months post-intervention (75% proforma usage), whereas Alamri et al*.* observed comparatively higher compliance with documentation criteria up to two years post-intervention (> 80% completion across most documentation criteria) [27, 28]. In contrast, Krishnamohan et al*.* observed a mild decrease in compliance with documentation criteria in the three-month period post-intervention, from 79 to 72%.

## Discussion

Surgical rounding is an important aspect of perioperative care, with deficiencies in ward round communication and documentation associated with poorer patient outcomes [3, 5, 28, 38, 44]. This systematic review summarised the results from 28 studies which implemented different surgical ward round interventions to improve perioperative care, with significant improvements shown in the quality of documentation and communication during ward rounds. Studies implementing active ward round breaks, telerounding, and digital patient records demonstrated positive feedback from staff and patients. Checklists or proformas were used most frequently to guide ward rounds and were typically associated with significant improvements in ward round documentation. This is consistent with advice from both The Royal College of Physicians and The Royal College of Nursing, who emphasise the utility of checklists in reducing medical errors, establishing rigorous documentation, and promoting cost-effective strategies for punctual discharge [47]. Other studies have also demonstrated the benefit of checklists for patient documentation and communication [3, 28].

Few studies measured the impact of ward round interventions on patient morbidity and mortality. However, implementation of ward round checklists led to significant reductions in prescribing errors and critical errors related to the management of postoperative complications [1, 3]. It was not possible to determine which of these factors were associated with the greatest impact on patient outcomes. This is an important consideration given that quality improvement strategies targeted at ‘high impact’ interventions are likely to result in disproportionately greater improvements in patient morbidity and mortality. The lack of assessment of clinically meaningful outcomes is a missed opportunity in context of the work required to develop ward round tools.

Subjective improvements in staff and patient communication were demonstrated with the use of checklists or proformas during the surgical ward round [13, 27, 29, 32, 34]. Documentation during the ward round is an important means of communication between clinical teams, with improvements in communication shown to mitigate medical errors and improve patient safety and outcomes [2, 9, 38, 48, 49]. Future studies should aim to develop more objective measures of staff and patient communication to improve assessment of different perioperative quality improvement strategies.

Only three studies assessed longitudinal outcomes of their ward round interventions over time [3, 27, 28]. Any successful ward round intervention should be simple and practical to implement, and consider all parties involved in order to achieve long-term engagement and compliance [14, 30, 36]. Further study into the durability of different perioperative ward round interventions would aid understanding of how improvement is maintained, which factors contribute to long-term adherence, and what strategies may overcome barriers of implementation.

Timing and efficiency of the surgical ward round is another consideration, with some staff apprehension about the extra time required to complete quality improvement interventions [50]. However, evidence regarding the impact of perioperative interventions on ward round timing is conflicting. Use of mobile tablets during the ward round led to a significant reduction in the ward round duration, suggesting that digitalisation may reduce time consuming activities such as finding physical notes or leaving the bedside to view investigation results [37]. Some studies found that checklists reduced ward round time [21, 38] possibly because they provided a set ward round structure. This could be useful as checklists provide a comprehensive ward round agenda, thus reducing the risk of omitting important considerations.

There are several limitations to this review. Data were derived from single-centre studies, with short follow-up durations and infrequent reporting of clinically relevant patient outcomes (e.g. morbidity and mortality). The predominance of observational studies (~ 90% of studies) also introduces considerable selection and confounding bias, limiting the reliability of our conclusions. Most studies also used non-validated questionnaires to measure staff and patient satisfaction. The heterogeneity in outcomes and reporting of data between studies made it difficult to perform meaningful quantitative analyses. In addition, potential impacts of the Hawthorne effect (the phenomenon where an individual may alter or change their behaviour when they are aware of being observed) on outcomes was not accounted for in any of the studies [47], which could be contributing to poor long-term durability of some interventions. Finally, ward round checklists or proformas were the most frequently studied intervention, which possibly relates to their relative ease of development and implementation. Thus, the impact of intervention selection bias could not be determined, despite a systematic and broad search of the surgical literature being performed. This suggests that barriers such as the lack of funding and/or resources may exist, ultimately inhibiting transformative interventions from being trialled in the setting of a surgical ward round.

Future research into the impact of different perioperative interventions should focus on larger patient cohorts, longitudinal follow-up of results, and objectively assessing for improvements in clinical outcomes via audit. The clinical and organisational framework for an optimal ward round are also important considerations, with key aspects being communication, early detection of complications, resilience to staff changes, staff well-being, efficiency, and regular auditing of ward round practices.

## Conclusion

Different types of ward round interventions have been implemented to improve the quality of patient care during the perioperative period. Use of checklists or proformas, telerounding, mobile tablet use, electronic patient records, a ‘surgeon of the week’ ward rounding system, as well as the introduction of active breaks during ward rounds have been associated with improvements in ward round documentation, communication, and satisfaction among staff and patients. Future studies should specifically investigate whether these different interventions are feasible to maintain in the long term, and their impact on clinically relevant outcomes such as patient morbidity and mortality.

### Supplementary Information

Below is the link to the electronic supplementary material.

Supplementary file1 (PDF 672 KB)
