# Supplementary material for: Interventions to Improve Patient Care on Surgical Ward Rounds: A Systematic Review
Source: World J Surg. 2023 Oct 19;47(12):3159–74. doi: 10.1007/s00268-023-07221-z (PMC10694108; doi:10.1007/s00268-023-07221-z)
Supplement: Supplementary file 1 — Supplementary file1 (PDF 672 KB) [file 268_2023_7221_MOESM1_ESM.pdf]

Improving patient care on surgical ward rounds: A systematic review

Reuben He, Sameer Bhat, Chris Varghese, Jeremy Rossaak, Celia Keane, Wal Baraza,

Cameron I. Wells

TABLE OF CONTENTS

Supplementary Appendix S1: *Preferred Reporting Items for Systematic reviews and Meta-Analyses (PRISMA) checklist*

Supplementary Appendix S2: *Exemplar search string when applied to the MEDLINE (OVID) database*

Supplementary Figure S1: *Quality assessment of prospective cohort studies using the Risk of Bias In Non-Randomized Studies of Interventions (ROBINS-I) tool*

Supplementary Appendix S3: *Quality appraisal of included retrospective cohort studies using the Joanna Briggs Institute Critical Appraisal Checklist*[23]

Supplementary Figure S2: *Summary risk of bias assessment results derived using the Cochrane Collaboration's Risk of Bias 2.0 (ROB2) tool*[24]

Supplementary Figure S3: *Risk of bias in each included randomized controlled trial based on the five domains using the Cochrane Collaboration's Risk of Bias (ROB2) tool*[24]

Supplementary Appendix S4: *Example of a ward round checklist*[9]

Supplementary Appendix S5: *Summary of findings and limitations of included studies grouped by theme of intervention*

Supplementary Appendix S1. Preferred Reporting Items for Systematic reviews and Meta-Analyses (PRISMA) checklist

Section and Topic	Item #	Checklist item	Location where item is reported
TITLE			
Title	1	Identify the report as a systematic review.	1
ABSTRACT			
Abstract	2	See the PRISMA 2020 for Abstracts checklist.	2-3
INTRODUCTION			
Rationale	3	Describe the rationale for the review in the context of existing knowledge.	4
Objectives	4	Provide an explicit statement of the objective(s) or question(s) the review addresses.	4-5
METHODS			
Eligibility criteria	5	Specify the inclusion and exclusion criteria for the review and how studies were grouped for the syntheses.	6-7
Information sources	6	Specify all databases, registers, websites, organisations, reference lists and other sources searched or consulted to identify studies. Specify the date when each source was last searched or consulted.	6, Appendix S2

Search strategy	***7***	***Present the full search strategies for all databases, registers and websites, including any filters and limits used.***	***6, Appendix S2***
Selection process	***8***	***Specify the methods used to decide whether a study met the inclusion criteria of the review, including how many reviewers screened each record and each report retrieved, whether they worked independently, and if applicable, details of automation tools used in the process.***	***7***
Data collection process	***9***	***Specify the methods used to collect data from reports, including how many reviewers collected data from each report, whether they worked independently, any processes for obtaining or confirming data from study investigators, and if applicable, details of automation tools used in the process.***	***7-8***
Data items	***10a***	***List and define all outcomes for which data were sought. Specify whether all results that were compatible with each outcome domain in each study were sought (e.g. for all measures, time points, analyses), and if not, the methods used to decide which results to collect.***	***7-8***
	10b	***List and define all other variables for which data were sought (e.g. participant and intervention characteristics, funding sources). Describe any assumptions made about any missing or unclear information.***	***7-8***
Study risk of bias assessment	***11***	***Specify the methods used to assess risk of bias in the included studies, including details of the tool(s) used, how many reviewers assessed each study and whether they worked independently, and if applicable, details of automation tools used in the process.***	***8***
Effect measures	***12***	***Specify for each outcome the effect measure(s) (e.g. risk ratio, mean difference) used in the synthesis or presentation of results.***	***N/A***
Synthesis methods	***13a***	***Describe the processes used to decide which studies were eligible for each synthesis (e.g. tabulating the study intervention characteristics and comparing against the planned groups for each synthesis (item #5)).***	***8-9***
	13b	***Describe any methods required to prepare the data for presentation or synthesis, such as handling of missing summary statistics, or data conversions.***	***N/A***
	13c	***Describe any methods used to tabulate or visually display results of individual studies and syntheses.***	***8-9***

	13d	***Describe any methods used to synthesize results and provide a rationale for the choice(s). If meta-analysis was performed, describe the model(s), method(s) to identify the presence and extent of statistical heterogeneity, and software package(s) used.***	***8-9***
	13e	***Describe any methods used to explore possible causes of heterogeneity among study results (e.g. subgroup analysis, meta-regression).***	***N/A***
	13f	***Describe any sensitivity analyses conducted to assess robustness of the synthesized results.***	***N/A***
Reporting bias assessment	*14*	***Describe any methods used to assess risk of bias due to missing results in a synthesis (arising from reporting biases).***	***N/A***
Certainty assessment	*15*	***Describe any methods used to assess certainty (or confidence) in the body of evidence for an outcome.***	***N/A***
RESULTS			
Study selection	*16a*	***Describe the results of the search and selection process, from the number of records identified in the search to the number of studies included in the review, ideally using a flow diagram.***	***10, Figure 1***
	16b	***Cite studies that might appear to meet the inclusion criteria, but which were excluded, and explain why they were excluded.***	***Figure 1***
Study characteristics	*17*	***Cite each included study and present its characteristics.***	***10, Table 1***
Risk of bias in studies	*18*	***Present assessments of risk of bias for each included study.***	***10-11, Figure S1, Appendix S3, Figures S2-3***
Results of individual studies	*19*	***For all outcomes, present, for each study: (a) summary statistics for each group (where appropriate) and (b) an effect estimate and its precision (e.g. confidence/credible interval), ideally using structured tables or plots.***	***11-15, Table 2, Appendices S4-5***

Results of syntheses	***20a***	***For each synthesis, briefly summarise the characteristics and risk of bias among contributing studies.***	***N/A***
	20b	***Present results of all statistical syntheses conducted. If meta-analysis was done, present for each the summary estimate and its precision (e.g. confidence/credible interval) and measures of statistical heterogeneity. If comparing groups, describe the direction of the effect.***	***N/A***
	20c	***Present results of all investigations of possible causes of heterogeneity among study results.***	***N/A***
	20d	***Present results of all sensitivity analyses conducted to assess the robustness of the synthesized results.***	***N/A***
Reporting biases	***21***	***Present assessments of risk of bias due to missing results (arising from reporting biases) for each synthesis assessed.***	***N/A***
Certainty of evidence	***22***	***Present assessments of certainty (or confidence) in the body of evidence for each outcome assessed.***	***N/A***
DISCUSSION			
Discussion	***23a***	***Provide a general interpretation of the results in the context of other evidence.***	***16-21***
	23b	***Discuss any limitations of the evidence included in the review.***	***19-20***
	23c	***Discuss any limitations of the review processes used.***	***19-20***
	23d	***Discuss implications of the results for practice, policy, and future research.***	***20***
OTHER INFORMATION			

Registration and protocol	*24a*	*Provide registration information for the review, including register name and registration number, or state that the review was not registered.*	*6*
	24b	*Indicate where the review protocol can be accessed, or state that a protocol was not prepared.*	*6*
	24c	*Describe and explain any amendments to information provided at registration or in the protocol.*	*N/A*
Support	*25*	*Describe sources of financial or non-financial support for the review, and the role of the funders or sponsors in the review.*	*1*
Competing interests	*26*	*Declare any competing interests of review authors.*	*1*
Availability of data, code and other materials	*27*	*Report which of the following are publicly available and where they can be found: template data collection forms; data extracted from included studies; data used for all analyses; analytic code; any other materials used in the review.*	*26*

Supplementary Appendix S2. Exemplar search string when applied to the MEDLINE (OVID) database

The database search was conducted on October 7, 2022.

Ovid MEDLINE (R) Epub Ahead of Print, In Process & Other Non-Indexed Citations, Ovid MEDLINE (R) Daily, and Ovid MEDLINE (R) 1946-Present:

1. Surg*
2. Cardiothoracic
3. Cardiac
4. Thoracic
5. Neurosurg*
6. Ortho*
7. Otolaryngology
8. Otorhinolaryngology
9. Head and neck
10. Paediatric surg*
11. Pediatric surg*
12. Plastic*
13. Urolog*
14. Vascular
15. Reconstruct*.mp
16. 1 OR 2 OR 3 OR 4 OR 5 OR 6 OR 7 OR 8 OR 9 OR 10 OR 11 OR 12 OR 13 OR 14 OR 15
17. Ward
18. Bedside
19. Teaching
20. Healthcare
21. Health care
22. Medical
23. Surg*
24. Morning
25. Daily
26. Attending
27. Consultant
28. Registrar
29. Resident
30. Patient
31. Patient care
32. 17 OR 18 OR 19 OR 20 OR 21 OR 22 OR 23 OR 24 OR 25 OR 26 OR 27 OR 28 OR 29 OR 30 OR 31

33. round*.mp
34. 32 adj 33
35. 16 AND 34
36. limit 35 to English language

#mp=title, abstract, original title, name of substance word, subject heading word, floating sub-heading word, keyword heading word, organism supplementary concept word, protocol supplementary concept word, rare disease supplementary concept word, unique identifier, synonyms.

Supplementary Figure S1. Quality assessment of prospective cohort studies using the Risk of Bias In Non-Randomized Studies of Interventions (ROBINS-I) tool.[22]

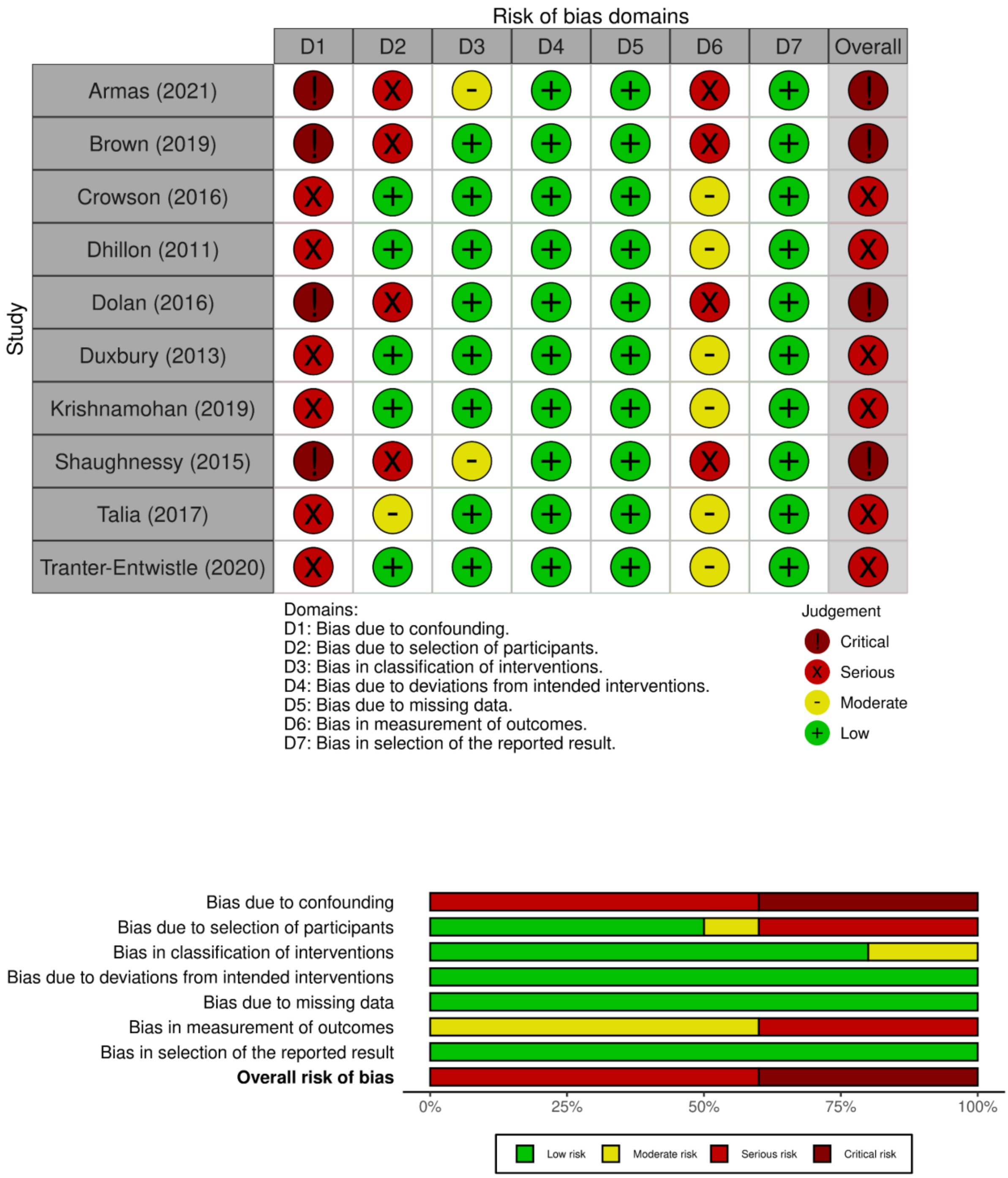

Supplementary Appendix S3. Quality appraisal results of included retrospective cohort studies using the Joanna Briggs Institute Critical Appraisal Checklist.[23]

First author (year)	Appraisal Question										
	**1**	**2**	**3**	**4**	**5**	**6**	**7**	**8**	**9**	**10**	**11**
Abbas (2016)	Y	Y	Y	N	N	N/A	Y	Y	N	N	Y
Al-Mahrouqi (2013)	Y	Y	Y	N	N	N/A	Y	Y	N	N	Y
Alamri (2016)	Y	Y	Y	N	N	N/A	Y	Y	Y	N/A	Y
Alazzawi (2016)	Y	Y	Y	N	N	N/A	N/A	Y	Y	N/A	U
Baker (1986)	Y	Y	Y	N	N	N/A	Y	Y	Y	N/A	U
Banfield (2018)	Y	Y	Y	N	N	N/A	U	Y	Y	N/A	U
Blucher (2014)	Y	Y	Y	N	N	N/A	Y	Y	Y	N/A	Y
Byrnes (2009)	Y	Y	Y	N	N	N/A	Y	Y	Y	N/A	Y
Chaudary (2022)	Y	Y	Y	N	N	N/A	Y	Y	Y	N/A	Y
Gilliland (2018)	Y	Y	Y	N	N	N/A	Y	Y	Y	N/A	U
Koumoullis (2020)	Y	Y	Y	N	N	N/A	Y	Y	Y	N/A	U
Ng (2018)	Y	Y	Y	N	N	N/A	Y	N	Y	N/A	Y

Pitcher (2016)	Y	Y	Y	N	N	N/A	Y	Y	Y	N/A	Y
Vukanic (2021)	Y	Y	Y	N	N	N/A	Y	Y	Y	N/A	U
Yorkgitis (2018)	Y	Y	Y	N	N	N/A	Y	Y	Y	N/A	Y

N, No; N/A, Not Applicable; U, Unclear; Y, Yes.

Appraisal questions:

1. Were the two groups similar and recruited from the same population?
2. Were the exposures measured similarly to assign people to both exposed and unexposed groups?
3. Was the exposure measured in a valid and reliable way?
4. Were confounding factors identified?
5. Were strategies to deal with confounding factors stated?
6. Were the groups/participants free of the outcome at the start of the study (or at the moment of exposure)?
7. Were the outcomes measured in a valid and reliable way?
8. Was the follow up time reported and sufficient to be long enough for outcomes to occur?
9. Was follow up complete, and if not, were the reasons to loss to follow up described and explored?
10. Were strategies to address incomplete follow up utilized?
11. Was appropriate statistical analysis used?

Supplementary Figure S2. Summary risk of bias assessment results derived using the Cochrane Collaboration's Risk of Bias 2.0 (ROB2) tool.[24]

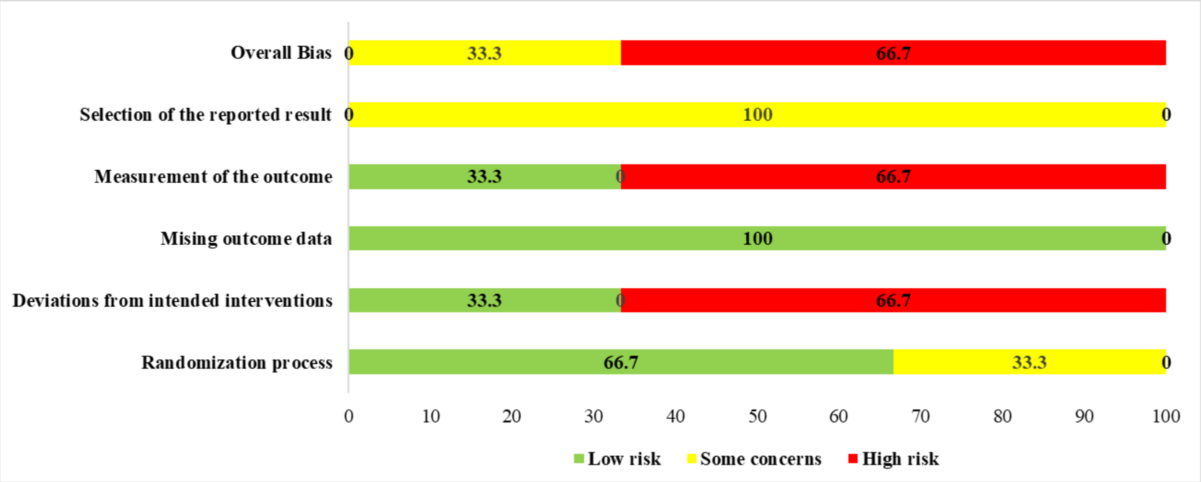

Supplementary Figure S3. Risk of bias in each included randomized controlled trial based on the five domains using the Cochrane Collaboration's Risk of Bias (ROB2) tool.[24]

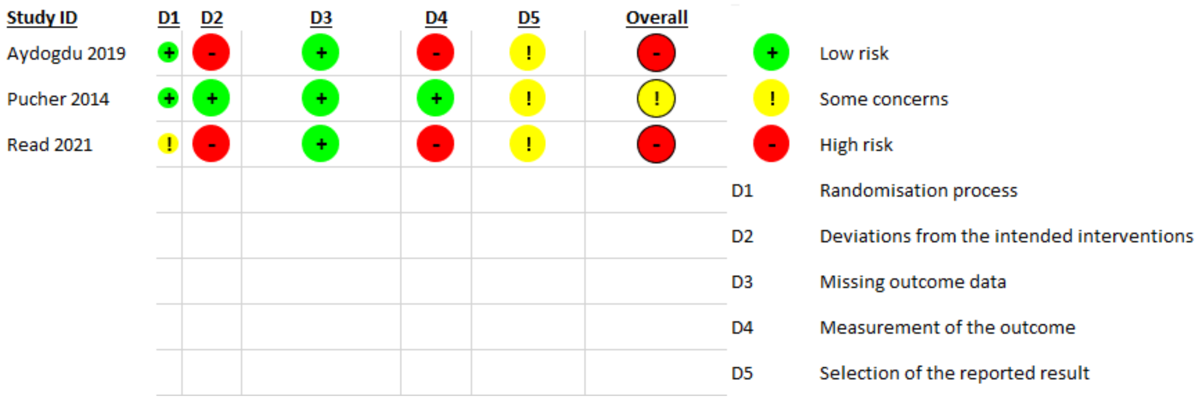

Supplementary Appendix S4. Example of a ward round checklist[9]

Post-Acute Ward Round

Surgeon/Doctor:
Date & Time: / / ____

Patient Label

1 BEFORE REVIEW

Confirm:
- Notes reviewed
- Investigations reviewed
- Hand hygiene

REVIEW

Patient & Nursing Concerns

History

Examination EWS: **Bloods/Imaging**

2 AFTER REVIEW

- Confirm hand hygiene
- Discuss antibiotics/therapy
- Discuss thromboprophylaxis
- Discuss fluids
- Discuss nutrition
- Review catheters & cannulas

Impression/Problem List **Clinical concern:** Low Medium High

Plan

3 BEFORE LEAVING

Confirm:
- Plan (with team)
- All above documentation complete
- Hand hygiene

Name
Signature
Contact

Supplementary Appendix S5. Summary of findings and limitations of included studies grouped by theme of intervention

Checklist/proforma

First author (year)	Intervention	Participants	Main findings	Limitations
Al-Mahrouqi (2013)	Post-acute ward round pro forma / checklist	General surgery inpatients<br>108 patients (pre-intervention)<br>103 patients (post-intervention)	Improvement in documentation of time and date (37% vs 72%) and impression (40% vs 61%); improvement in documentation of dietary plan when pro forma filled out (78/103 patients, 76%); no statistically significant impact on nurse certainty of dietary plan and number of times needed to contact surgical teams	Contamination from nurses discussing study; lack of complete documentation on post-acute consultant ward round; low maintenance of intervention (75% pro forma usage 6 months post-intervention); poor survey response rate
Alamri (2016)	Ward round checklist / pro forma	General surgery inpatients<br>103 patients, 479 proforma stickers (post-intervention)	Most fields in pro forma documented to adequate level (>80%) 2 years post-intervention; problematic fields were dietary plans, diagnosis, national health index number, estimated date of discharge and patient first name; notes of patients on outlying ward had significantly fewer pro forma per day than home -ward (0.71 vs 1.21 pro forma per day)	Timing bias, ‘snapshot’ vs longitudinal study; lack of exploration of freehand notes to identify reasons for pro forma documentation deficiency
Alazzawi (2016)	Ward round pro forma / checklist	Trauma and orthopaedics inpatients<br>20 patients (pre-intervention)<br>20 patients (post-intervention)	Significant increases in documentation of diagnosis and management, objective assessments (excluding observations noted), and logistics; 10 members of staff all preferred pro forma vs standard care due to ease of reading and clarity of information	Effect on clinical assessment and patient care not measured; unblinded study; large amount of undocumented clinical activity

Banfield (2018)	Post-acute ward round pro forma / checklist	General surgery inpatients<br>31 patient notes (pre-intervention)<br>27 patient notes (cycle 2)<br>26 patient notes (cycle 3.1)<br>20 patient notes (cycle 3.2)	Improvement in documentation of VTE assessment, fluids, observations and investigations post-intervention; improved weekend documentation in all categories except length of stay; junior team members found that checklist improved understanding of diagnosis, management plan, and ward round effectiveness	Small sample size; reduced checklist access for outlying patients
Blucher (2014)	Ward safety pro forma / checklist	General surgery inpatients<br>49 patients (pre-intervention)<br>51 patients (post-intervention)	Overall significant improvement in introduction phase components of checklist (31% vs 52%); overall significant improvement in time-out phase components (37% vs 45%); overall significant improvement in actions phase components (48% vs 56%)	Small sample size; no standardisation of time-out phase components in checklist; Effect on clinical assessment and patient care not measured
Brown (2019)	Surgical communication check sheet / pro forma	Trauma and orthopaedics inpatients<br>170 patients (pre-intervention)<br>111 patients (post-intervention)	Between cycle 1 and 3: reduction in percentage of patients with unanswered questions (21.8% vs 16.7%), reduction in number of patients unsure why a test was done (25.9% vs 12.7%), improvement in average understanding of management plan (64.7% to 83.3%)	Study unblinded; reduced sample size (survey compliance issues)

Byrnes (2009)	Ward round checklist / pro forma	Surgical and trauma ICU inpatients<br>53 patients, 583 assessments (pre-intervention)<br>61 patients, 671 assessments (post-intervention)	Verbal consideration of domains improved from 90.9% to 99.7% after intervention; bedside consideration improved for use of DVT prophylaxis, stress ulcer prophylaxis, oral care for ventilated patients, electrolyte repletion, initiation of physical therapy, and documentation of restraint orders; checklist resulted in >2 fold increase in transferring patients out of ICU on telemetry and initiation of physical therapy compared to pre-intervention	Contamination bias in consideration phase (as checklist was optional for both groups); observer bias; no quantifiable data for some domains on checklist (e.g. tracheostomy protocol, need for central venous catheter, nutrition); questions about longitudinal checklist maintenance
Dhillon (2011)	Ward round checklist	General surgery, vascular surgery, plastic surgery, and neurosurgery patients<br>53 patients (pre-intervention)<br>34 patients (post-intervention)	Improvement in percentage adherence to the Good Surgical Practice Guidelines ‘across the board’ 55% pre- vs 91% post-intervention); significant improvement in documentation across all areas measured	Did not measure effect on morbidity and mortality; Hawthorne effect;
Dolan (2016)	Post-take ward round checklist / pro forma	Acute surgical inpatients<br>50 patients (pre-intervention)<br>47 patients (post-intervention)	Improvement in documentation compliance across multiple categories: patient name/identification number (96% to 100%), subjective findings (84% to 100%), objective findings (48% to 100%), plan (98% to 100%), signature (96% to 100%), grade (92% to 100%), clinical impression/diagnosis (30% to 98%), diet status (16% to 83%), discharge decision (16% to 66%), discharge planning (20% to 40%)	Small sample size; unblinded (Hawthorne effect)

Duxbury (2013)	Post-take ward round checklist / pro forma	Trauma and orthopaedics inpatients<br>50 patients (pre-intervention)<br>50 patients (post-intervention)	Number of patients not seen on PTWAR decreased (28% to 18%); improvements in documentation of following categories: date (97% to 100%), time (83% to 88%), consultant on-take (81% to 96%), clinician leading ward round (81% to 88%), presenting complaint (22% to 90%), management plan (97% to 98%), signature (86% to 100%), grade (50% to 90%), contact details (75% to 85%)	Small sample size; poor compliance to checklist during weekends, unblinded
Gilliland (2018)	Ward round template / checklist	Urology inpatients<br>14 patient notes (cycle 1)<br>17 patient notes (cycle 2)<br>14 patient notes (cycle 3)	Documentation of baseline measurements improved significantly following introduction of standardised ward round template, notably for: documentations of VTE risk assessment (14% to 92%) and antibiotic stewardship (0% to 100%), and use of the treatment escalation plan form (29% to 78%)	Small sample size; patient outcomes not measured, assumption of association between improved documentation and improved patient outcomes
Koumoullis (2020)	Surgical Tool for the Assessment of Rounds (STAR) checklist / pro forma	Plastic surgery inpatients<br>42 patients (pre-intervention)<br>103 patients (post-intervention)	Checklist implementation improved STAR completion rate (47% to 70% to 88%); unsolicited enthusiastic staff comments about ward round improvement after STAR implementation	Hawthorne effect, weekend exclusion, seasonal patient variation
Krishnamohan (2019)	Ward round checklist	Urology and vascular surgery inpatients<br>72 case notes (pre-intervention)<br>61 case notes (post-intervention)	Overall documentation of six checklist parameters improved following implementation (26% to 79%); largest parameter increase was documentation of fluid balance (8% to 76%); 3 month follow-up showed maintenance of 72% documentation compliance	Checklist reporting bias; quality of documentation not assessed; Hawthorne effect; relevance to patient outcomes not measured

Ng (2018)	Ward round sticker / checklist	General surgery inpatients<br>109 ward round entries (pre-intervention)<br>147 ward round entries (post-intervention)	Baseline checking of drug chart, intravenous fluid chart, analgesia, antiemetic, enoxaparin, thromboembolic deterrents ranged from 0% to 6%, all significantly improved with implementation of ward round stickers	Relevance to patient outcomes not measured; data for outlying patients not collected; Hawthorne effect
Pitcher (2016)	Ward round checklist	General surgery inpatients<br>132 patient interactions (pre-intervention)<br>182 patient interactions (post-intervention)	Significant improvement in the consideration of the majority of checklist criteria (bedside consultation, patient safety, bedside charts, planning, documentation and summary) following intervention (P<0.05 for all criteria)	Hawthorne effect (surgical team blind to nature of observations but were aware that observation was being conducted)
Pucher (2014)	Ward round checklist	General surgery trainees<br>10 trainee registrars (no checklist)<br>10 trainee registrars (checklist)	Intervention group subjects using checklist had significantly fewer critical errors compared with controls (median(i.q.r.) 0(0-0) vs 60(40-73)%; intervention group had improved patient management and non-technical skills between baseline and final ward rounds, whereas controls did not (P<0.05 for all categories); subjective ease of checklist use	Did not measure checklist use for medical staff outside of surgical trainees; single centre study; did measure maintenance of checklist over time;

Read (2021)	Ward round checklist	Unspecified surgical inpatients 68 patients (pre-intervention) 56 patients (post-intervention)	Overall percentage of checklist items endorsed increased significantly after intervention (64.8% to 70.0%); statistically significant improvements for following categories: patients knowing their diagnosis, day plan, medication chart review, and observation chart review	Small sample size; patient could not compare standard vs checklist-implemented ward rounds as only subjected to one or the other; poor compliance with checklist completion from surgical teams; Hawthorne effect
Shaughnessy (2015)	Ward round checklist	Cardiothoracic surgery inpatients 222 patients (pre-intervention) 83 patients (post-intervention)	97% of nurses agreed that verbal checklist summarising improved clarity and 90% felt it improved patient care; 87% of MDT respondents noticed improvement in bedside nurse attendance during ward round; ward round checklist reduced omissions but patient communication required further improvement	Patient understanding of ward round not measured; large variation in pre- vs post-checklist observation numbers – time limitation of post-audit; difficulty enforcing nurse checklist review compliance
Talia (2017)	Ward round checklist	Orthopaedics patients 132 patient encounters (pre-intervention) 68 patient encounters (post-intervention)	After introduction of the checklist, documentation of surgical details (38.6% to 85.3%), fasting status (9.1% to 70.6%), VTE prophylaxis (6.8% to 92.6%), and weight-bearing status (11.4% to 83.8%) improved (all $P<0.0001$); VTE prophylaxis discussion improved from 9.8% to 45.6%	Variation in pre- and post-checklist sample sizes; did not measure impact on patient outcomes

Tranter-Entwistle (2020)	Ward round checklist	Vascular surgery inpatients<br>60 patient consultations (pre-intervention)<br>89 patient consultations (cycle 1)<br>84 patient consultations (cycle 2)	20/21 ward round quality indicators showed statistically significant improvement after checklist implementation, notably observation chart review (20% to 75% to 81%), drug chart review (10% to 54% to 78.6%), and anticoagulation/antiplatelet treatment (32% to 61% to 58.1%); mean consultation time per patient did not increase post-intervention; all subjective measures showed significant improvement post-intervention	Lack of external checklist validation; single centre; single observer; no measure of impact on patient outcomes
Yorkgitis (2018)	Laboratory tests and chest X-ray imaging section on daily ICU checklist	SICU inpatients<br>155 patients (pre-intervention)<br>152 patients (post-intervention)	No statistical reduction in laboratory tests or chest x-ray imaging ordered per day after checklist implementation	Checklist fatigue; checklist not reviewed daily;
Vukanic (2021)	Ward round pro forma	Orthopaedics inpatients<br>30 patients (pre-intervention)<br>30 patients (post-intervention)	After pro-forma introduction, average documentation criteria fulfilment percentage increased (0% to 86%); maintenance was 75% criteria fulfilment after 2 months	Small sample size; baseline data collected on single day

Technology

First author (year)	Intervention	Participants	Main findings	Limitations
Aydogdu (2019)	Additional tele-rounding on patients following surgery	Urology inpatients<br>40 patients (standard rounding)<br>40 patients (telerounding)	Mean time of preoperative telerounding visits was 3.65±0.59 minutes. Mean time of telerounding visits on the postoperative 1st and 2nd days was 3.80±0.62 and 2.9±0.91 respectively; visual analogue scale for surgeon satisfaction rate for telerounding was 91±11.2; patients expressed satisfaction rate of 72.5%	Small sample size; survey not validated; confounding variable of increased surgeon availability vs telerounding itself
Chaudary (2022)	Digital / electronic patient records	Trauma and orthopaedics staff surveys<br>44 staff (14 nurses, 13FY, 8 registrars, 5 SMOs, 4 other clinical support workers)	Overall staff satisfaction score significantly higher for electronic methods vs paperwork for all four survey questions combined; individually, electronic score higher for 'opportunity to learn images in ward round), comparable results for 'educational useful of ward round' and 'typing time affecting learning time'; electronic record more effective for adherence to guidelines across multiple categories	Small sample size; single centre study; participants not equally distributed across qualification and experience; data documentation not cross-checked with paper documentation to determine validity
Crowson (2016)	Mobile tablet use during ward rounds	Otorhinolaryngology staff<br>13 otolaryngology residents survey answers	Time for inpatient rounding shorter with use of tablets (p=0.037); non-significant trend for number of times a resident had to leave rounds to look up a clinical query on a computer; subjective feedback that tablet use facilitated more detailed and faster transfer of information and improved ease of documentation in the medical record; tablet use allowed more time directly involved in rounds in 70% of responses; tablets suggested to improve morale in 80% of survey responses	Small sample size; subjective measurements (survey responses); potential financial subsidy of tablets may bias survey results; 'complicated' patients as confounding for rounding time not accounted for; low (77%) post-intervention survey response rate in context of small sample size

Personnel

First author (year)	Intervention	Participants	Main findings	Limitations
Abbas (2016)	'Surgeon of the week' rounding system	Paediatric surgery inpatients 2,356 inpatient encounters (pre-intervention) 2,837 inpatient encounters (post-intervention)	Total number of safety complaints decreased after intervention (37 pre- vs 27 post-); work relative units increased by 8%; nonoperative billing increased by 15%; employee satisfaction (55% vs 83%) and parental satisfaction increased (33% vs 75%)	Recall bias in surveys; patient volume variation affecting safety and relative unit outcomes; subjective patient satisfaction measures; generalisability to all surgical specialties
Baker (1986)	Presence of a radiologist during ward rounds	General surgery inpatients 721 patients (pre-intervention) 765 patients (post-intervention)	Reduction in number of nuclear medicine scans, ultrasound scans, body CT scans, barium enemas, and upper GI series obtained post-intervention; increase in number of abdominal plain films obtained post-intervention; average length of stay decreased from 21.4 to 18.4 days post-intervention	Time period bias and therefore current relevance of study; no attempt to compare disease types and severity in experimental vs control groups; cost consciousness and fiscal restraint imperatives confounding length of stay

Wellbeing

First author (year)	Intervention	Participants	Main findings	Limitations
Armas (2021)	Active / scheduled breaks during ward rounds	SICU rounding team surveys 8-12 members at a time, 30 survey responses	Majority of participants thought active breaks relieved stress (27/30, 90%), promoted wellness (29/30, 96.7%), and improved team morale (29/30; 93.1%); squats were favoured activity during breaks (17/30, 56.7%); active breaks were appropriate for the working environment (27/30, 90%); majority of respondents interested in maintaining active breaks as routine practice (90%)	Data not collected outside of morning ward rounds (uncertain impact at other times); study performed in one unit only at one centre; no pre-test survey for baseline comparison; Hawthorne effect; small sample size

SMO, senior medical officer; FY, foundation year; SICU, surgical intensive care unit; ICU, intensive care unit; CT, computerised tomography; VTE, venous thromboembolism; DVT, deep vein thrombosis; PTWR, post-take ward round; MDT, multi-disciplinary team
